# Career and life development intervention for non-engaged youth: Evaluating the Hong Kong Benchmarks (Community) Pilot Program

**DOI:** 10.3389/fpsyg.2023.1117251

**Published:** 2023-04-06

**Authors:** Steven Sek-yum Ngai, Chau-kiu Cheung, Qiushi Zhou, Lin Wang, Yuen-hang Ng, Winnie Pui-ching Leung, Elly Nga-hin Yu, Laing-ming Wong, Hon-yin Tang, Horace Cheuk-him Lee

**Affiliations:** ^1^Department of Social Work, The Chinese University of Hong Kong, Hong Kong, China; ^2^Department of Social and Behavioral Sciences, City University of Hong Kong, Hong Kong, China; ^3^Department of Social Work, Fudan University, Shanghai, China

**Keywords:** career and life development intervention, non-engaged youth, program evaluation, school-to-work transition, career-related competencies

## Abstract

In our study, aimed at examining the effectiveness and impact of the Hong Kong Benchmarks (Community) Pilot Program, a career and life development (CLD) intervention program targeting non-engaged youth (NEY) in Hong Kong, we employed a pretest–posttest quasi-experimental design to compare changes in career-related competencies between a pilot group (*N* = 289) and a comparison group (*N* = 160). We also conducted five focus group interviews with the leaders of nongovernmental organizations, social workers, NEY, parents, and employers to explore the program’s impacts on the CLD service provisions. Our quantitative results indicate that the piloting group showed greater improvement in two career-related competencies—youth career development competency and career and life development hope—than the comparison group. Meanwhile, our qualitative results suggest both the benefits and difficulties experienced by stakeholders in the program. The findings thus provide preliminary evidence of the Hong Kong Benchmarks (Community) Pilot Program’s positive impacts on NEY and other important stakeholders. The implications of expanding the existing program and theorizing the community-based benchmark approach are also discussed.

## 1. Introduction

The school-to-work transition refers to the period and process during which graduates leave school and enter the labor market (Phillips et al., 2002; Zamfir et al., 2018). It is a crucial phase for young people because it involves many important decisions concerning schooling, employment, and training (Ryan, 2001; Koen et al., 2012). During the transition, young people often need to make decisions that determine their later vocational outcomes and establish future career and life patterns (Ng and Feldman, 2007; Saks, 2018). When young people fail to adapt to the transition, they are at high risk of being stuck in unemployment, underemployment, or unstable employment (Koen et al., 2012; Park et al., 2020) and suffering from a myriad of adverse consequences, including low well-being (McKee-Ryan and Harvey, 2011), low life satisfaction (Kinicki et al., 2000), and increased physical and psychological strain (Cheng-Lai and Dorcas, 2011; Huegaerts et al., 2018). At the same time, research has also documented the importance of the school-to-work transition at the macro-socioeconomic level. For example, a high unemployment rate resulting from unsuccessful school-to-work transitions can lead to increased antisocial behaviors and thus increase social costs (Saks, 2018; Medvide et al., 2019). By contrast, an increased youth employment rate may eventually benefit national economic growth and, in turn, improve people’s living standards (Nilsson, 2019). As Morrison (2002) has summed up the situation, the school-to-work transition has great implications for both individuals and society.

Owing to economic globalization and advanced technology, however, the world of work has increasingly been characterized by uncertainty and unpredictability (Ngai et al., 2021, 2022) that makes the school-to-work transition more tortuous, longer, and less controllable than the direct, well-defined transition presumed to be available to previous generations (Ryan, 2001; Morrison, 2002; Allen and Van Der Velden, 2007). At present, young people are more likely than adults to encounter job mismatch and suffer from prolonged unemployment (Pavlova et al., 2018). Worse still, the COVID-19 pandemic has placed additional stress on youth (Dasgupta, 2022). In Hong Kong, for example, from February to April 2022, the unemployment rates for young people 15–20 and 20–29 years old were 17.2 and 7.7%, respectively, both of which outpaced the overall unemployment rate of 5.5% during the same period (Census and Statistics Department, 2022). Against that background, one group of youth in particular requires special attention: non-engaged youth (NEY), who are often considered to be more vulnerable than their peers due to multiple disadvantages in their school-to-work transitions and are at risk of becoming NEET youth (Ngai and Ngai, 2007; Mo and Lau, 2020). In Hong Kong, the various subgroups of NEY may include ethnic minority youth (i.e., young people of non-Chinese ethnicity in Hong Kong), unemployed youth (i.e., youth without paid employment), school dropouts (i.e., young people who left the formal education system early), youth offenders (i.e., young people who commit crimes and have been arrested), youth with special education needs (i.e., young people diagnosed with special education needs by helping professionals), and young mothers (i.e., young women who enter motherhood unprepared) (Ngai et al., 2023). In 2020, more than one in five (22.4%) youth worldwide were identified as NEY, all of whom face different disadvantages in the school-to-work transition (International Labour Organization, 2020).

Given high unemployment and the difficulty of career environments, providing young people with career intervention programs has been identified as an important strategy for bringing young people, especially NEY, back into employment, education, or training. In the past decade, various programs targeting NEY or NEET youth have indeed been developed and implemented worldwide. For example, in 2013, to reduce challenges facing NEY, the European Union called for member states to provide more education, apprenticeship, training, and employment opportunities under the Youth Guarantee Scheme (Maguire, 2015). In another case, the United Kingdom (UK) introduced the Youth Obligation Support Program in 2017 to support youth with improving their job searches, job applications, and interview skills. Meanwhile, New Zealand commenced the Youth Service: NEET in 2012 to support NEET youth with gaining higher-level qualifications able to enhance their employability. Beyond that, in South Korea, the Ministry of Employment and Labor launched the Employment Success Package Program in 2009, which addresses the problems experienced by NEET youth by supporting their career exploration, capacity enhancement, and job placement and by providing allowances (Park et al., 2020).

While career interventions or policies targeting NEY have mushroomed across the globe, most of them are limited to employment-focused job support and thus fail to fully respond to the complicated issues facing NEY (Park et al., 2020). On the one hand, the characteristics of NEY, including inactivity, passivity, lack of ambition, and low hope about their future life, have made them generally disenchanted with intervention programs that focus purely on skills training and vocational knowledge impartment (Ngai and Ngai, 2007; Mascherini et al., 2012; CLAP@JC, 2017; Caroleo et al., 2020). On the other, and as mentioned, the changing world of work has created various social barriers to conventional pathways to employment, which requires an increase in youth self-knowledge to navigate job opportunity structures. In that context, encouraging young people, especially NEY, to develop broader career-related competencies to steer their own career and life development (CLD) is crucial and urgently needed (Goldman-Mellor et al., 2016; Ngai et al., 2021).

In our study, we evaluated a pilot program, the Hong Kong Benchmarks (HKBM) for Career and Life Development (Community)—the HKBM (Community) Pilot Program, for short—aimed at helping NEY become navigators and active agents of their pathways to desired careers and lives by providing them with CLD interventions that can be benchmarked with world-class standards (see Sections 2.2 and 2.3 for details about the program). In particular, we examined both the program’s practical effectiveness in enhancing NEY’s career-related competencies to navigate transitions through education into productive employment (Ngai et al., 2021) and its impacts on different stakeholders, including leaders of nongovernmental organizations (NGOs), social workers, parents, and employers, all of whom play crucial roles in promoting those competencies among NEY.

## 2. Toward a benchmark approach to CLD interventions

### 2.1. Career-related competencies and CLD interventions

Career-related competencies refer to a range of capabilities needed to master the transition from education to productive employment and meaningful life. In addition to vocational skills and language proficiency, career-related competencies emphasize a strong sense of agency and commitment to work, the awareness to reflect on self, and the ability and motivation to actively explore career-related resources in a given environment (Akkermans et al., 2013; Ngai et al., 2021). In our study, we focused on two career-related competencies: career and life development hope (CLDH) and youth career development competency (YCDC).

On the one hand, CLDH, which refers to a positive motivational-cognitive-behavioral state toward the actualization of personal goals, has been highlighted as an indispensable resource for young people to get ahead in future careers and life (Snyder, 2002; Hong et al., 2020; Ngai et al., 2022). The positive motivational-cognitive-behavioral state consists of two components. The motivational component, *career and life development agency*, refers to initiating and sustaining motivation to achieve desired outcomes. It also refers to young people’s gaining agency in their CLD and their ability to initiate, execute, and sustain motivation toward achieving their desired career and life goals. By contrast, the cognitive-behavioral component, *career and life development pathways*, refers to the perceived cognitive and behavioral ability to generate multiple routes or pathways to achieve desired goals. The two components are thought to reinforce each other during the pursuit of goals and in acquiring positive outcomes in young people’s CLD (Ngai et al., 2022).

On the other hand, YCDC refers to youth’s ability to become active path navigators and change agents in their career pathways (Ngai et al., 2021). It consists of four major components: engagement, self-understanding, career and pathway exploration, and planning and career management (Ngai et al., 2021). Among them, engagement refers to young people’s ability to strengthen their connections with the community and thereby ignite or reignite their motivation for career building by engaging in activities and experiences related to career and life planning. By contrast, self-understanding represents young people’s ability to comprehend and reflect on their interests, strengths, and values, which allows them to pinpoint which career path(s) can help them to realize their potential. Next, career and pathway exploration denotes young people’s ability to explore and make sense of multiple pathways (e.g., further education, full-time employment, and workplace learning) that are compatible with their interests, strengths, and values. Last, planning and career management reflects youth’s ability to grasp a broader range of career-related competencies (e.g., skills to obtain and synthesize information and set realistic plans), make informed decisions, and implement steps in response to their decisions about pathways forward.

One way to enhance youth’s career-related competencies is by providing CLD-related intervention programs. The term CLD intervention is often used interchangeably with the term career guidance to refer to services intended to assist young people in making educational, training, and occupational choices, as well as managing their career and life pathways and thereby actively achieving established goals in their social contexts (Leung, 2005; Mann et al., 2020). In the past several decades, CLD interventions have been widely used by schools in different countries and territories to cultivate young students with career-related competencies (Hooley and Rice, 2019). Evidence suggests that CLD interventions can generate positive outcomes in terms of young people’s career readiness, career decidedness, intentions to find a job, self-reliance, and socioeconomic benefits (Chen, 2011; Holman, 2014; Hirschi, 2018; Park et al., 2018, 2020). CLD interventions are promising because they consider young people’s diverse needs, thereby enabling them to generate multiple pathways to approach desired careers and life. They are limited, however, because they are often designed and implemented in diverse ways, which raises the vital question of what constitutes good CLD interventions (Holman, 2014).

### 2.2. Development of a benchmark approach to CLD intervention

To address that question, a benchmark approach to CLD intervention has been developed. Denoting both measurable global standards and a process of self-improvement, the benchmark approach specifies what good CLD practices would look like in the form of several benchmarks capturing different dimensions of quality CLD interventions (Holman, 2014). It also emphasizes the practical process through which CLD service providers (e.g., schools and NGOs) constantly evaluate and improve their CLD services against global standards (Holman, 2014). The benchmark approach has been embraced or popularized in schools in the United Kingdom and other regions worldwide (Careers and Enterprise Company, 2020; Gatsby Charitable Foundation, 2020). One of the most successful cases has been that of the Gatsby Benchmarks. In 2014, the Gatsby Charitable Foundation of the UK commissioned Professor Sir John Holman and his colleagues to pinpoint the essential elements of quality CLD interventions worldwide (Gatsby Charitable Foundation, 2015). Based on the evidence gathered from an international study conducted across Finland, Germany, Hong Kong, Ireland, the Netherlands, and Canada, Holman (2014) developed eight benchmarks that can determine world-class CLD interventions. The eight benchmarks were subsequently endorsed by the UK government as the basis for its strategy to implement CLD interventions in all schools in England (Department for Education, 2017). As that case shows, both governments and NGOs around the world have recognized the significance of CLD for achieving successful school-to-work transitions and are willing to design and implement benchmark-based CLD interventions in their own social and cultural contexts.

Although school, community, and the world of work are three common touchpoints for young people in the school-to-work transition, the use of the benchmark approach has long been concentrated in schools and rarely emerged in other settings. Taking the Gatsby Benchmarks as an example, whether in the UK or in other countries around the world, their application primarily focuses on schools (Holman, 2014). For an alternative, in 2021, a research team at the Chinese University of Hong Kong and partners from six experienced NGOs co-created the world’s first community version of benchmarks for quality CLD interventions. The effort also involved the participation of social workers, employers, and the creator of the Gatsby Benchmarks, Sir John Holman (CLAP@JC, 2022a). Expanding the impact of the benchmark approach from schools to communities is meaningful, chiefly because many NEY (e.g., young mothers and school dropouts) may have disengaged from school spheres long ago and because their daily activities more often take place in communities. As a result, the school-based benchmark approach may not seem inclusive to them. After all, the community can serve as an interface that bridges families, schools, centers for continued education, and the business world and generates benefits at the societal level (Park et al., 2020). NEY with a supportive community environment may receive personalized advice and support for identifying life goals, making career and life choices, and enhancing their connections with the community (e.g., *via* mentors and community partners) to access more job opportunities and broader career networks. In that light, conducting a pilot program under the HKBM (Community) framework can provide a direct, systematic response to NEY-related issues, help them to circumvent the risk of becoming NEET, and support them in becoming active navigators of their careers and life journeys.

The HKBM (Community) refers to a systematic self-improvement framework for NGOs to build quality CLD service provisions that can be benchmarked with global standards. The framework increases the number of benchmarks from the Gatsby Benchmarks from eight to ten, which together can be grouped into three parts: the core part, the youth-focused part, and the enabling environment part (see the Appendix; CLAP@JC E-learning Hub, 2022a). First, the core part is concerned with the NGOs that provide CLD interventions, especially whether they have established stable, visible policies and designated teams with professional competencies and leadership. Second, the youth-focused part, as its name indicates, places importance on youth’s personal needs, access to career-related information, the right to become masters of their pathways, and their reception of personal guidance to develop career roadmaps. Last, the enabling environment part considers broader environments beyond NGOs (e.g., parental support and meaningful encounters with the workplace), which can bridge NEY and a wide range of future career pathways. Notably, each benchmark can be broken down into a cluster of measurable criteria that enable CLD service providers to determine the extent to which their services and strategies measure up against a specific benchmark. With such the HKBM (Community) framework, NGO leaders and social workers can review their existing youth services quantitatively and qualitatively and, in turn, better assist NEY in attaining productive transitions to employment, education, and training.

### 2.3. The HKBM (Community) Pilot Program

Following the creation of HKBM (Community), the HKBM (Community) Pilot Program was launched under the territory-wide CLAP@JC project. CLAP@JC is a 10-year project and cross-sectoral support platform for CLD that aims at fostering a sustainable ecosystem by bringing together the education, community, and business sectors to facilitate the school-to-work transition for all youth (CLAP@JC, 2022b). To ensure fidelity in the program’s implementation, three rigorous mechanisms were created to launch, promote, and monitor the participating NGOs’ implementation of the program.

The first mechanism was to form the BM Core Committee as the steering body for the program, which was designed to consist of experts from the abovementioned research team at the Chinese University of Hong Kong and experienced practitioners from six participating NGOs. Next, the BM Core Committee created a checklist to be used by the BM Working Groups, which consisted of NGO leaders and social workers within each partner NGO, to help them to conduct self-assessments on their existing CLD services. The checklist also served as a guiding tool by covering sets of criteria under each benchmark that need to be fulfilled to achieve positive outcomes in changing the CLD ecosystem for youth. Last, the BM Working Groups provided supporting proof (e.g., worksheets, evaluation forms, debriefing notes, and orientation materials) for each criterion on the checklist, and the criteria were ranked at three levels according to the achievement of each: fully achieved, partly achieved, or emerging (CLAP@JC E-learning Hub, 2022b).

The BM Core Committee initiated the second mechanism to implement the three-day training program to train experienced social workers from the six participating NGOs to become BM Facilitators. Specifically, the training program provided a comprehensive understanding of the HKBM (Community) and its background, design, and principles of implementation to trainees. In addition, the program covered an in-depth explanation and discussion of the 10 benchmarks. Most important, after the training, the BM Facilitators were expected to fully understand the process and content of the checklist for the HKBM (Community) for the purpose of self-assessment and to have gained the skills to support the BM Working Groups in self-assessment to identify each NGO’s strengths and gaps in the implementation of the HKBM (Community). The BM Facilitators were also expected to have gained the knowledge to guide the NGOs in reflecting on the results of self-assessment and developing action plans for self-improvement. In other words, the role of the BM Facilitator was essential, for they were agents who imparted knowledge and supported the BM Working Groups’ members in gaining the skills and abilities in self-assessment by using the checklist, understanding the processes of the HKBM (Community), and offering constructive advice to BM Working Groups and NGOs.

For the third mechanism, the BM Core Committee, BM Facilitators, and BM Working Groups conducted review meetings. During the meetings, they discussed the rankings of each criterion of the benchmarks and shared their feedback regarding the strengths, suggested areas for improvement, and recommendations for improvement to the pilot NGOs regarding each benchmark. For example, in the evaluation results for the first benchmark, “A stable and visible career and life development policy,” the BM Core Committee and BM Facilitators suggested collecting and consolidating feedback from different stakeholders (e.g., employers, parents, and community partners) so that every party would help to enhance the policies to achieve smooth cooperation in improving CLD interventions. In addition, they encouraged the pilot NGOs to organize regular meetings with the stakeholders and collect feedback on CLD policies and challenges in implementing the benchmarks. Moreover, for the fifth benchmark, “Youth engagement and co-creation,” they advised strengthening NEY’s involvement in co-designing and co-implementing CLD activities with their responsible social workers. Meanwhile, for the ninth benchmark, “Meaningful encounters with further education opportunities,” they advised the NGOs to provide up-to-date information to the social workers about continuing education, especially information about continuing and higher education institutions, to enhance CLD consultation with NEY. Although the BM Core Committee provided recommendations to the pilot NGOs, the pilot NGOs were encouraged to formulate and implement their action plans for self-improvement according to their organizational needs and pace, which suggested an egalitarian and collaborative relationship between the BM Core Committee and the pilot NGOs. After the implementation of the action plans, the BM Facilitators held regular meetings with the BM Working Groups to review the strategies for the improvement of the HKBM (Community) in an effort to consolidate their practical wisdom and enhance their CLD interventions. In that way, the HKBM (Community) functioned as a framework geared toward self-improvement that enabled the NGOs to periodically measure their progress toward suitable CLD interventions against world-class standards. That assumption, however, needed to be supported by evaluations of programs with solid evidence.

The current research was conducted to evaluate the preliminary outcomes of the HKBM (Community) Pilot Program, particularly whether it, as claimed, could enhance the community-based CLD intervention and thus promote NEY’s school-to-work transition. Researchers have indicated that a sound program evaluation should focus not only on outcomes that can be quantitatively assessed but also on the operational aspects of the program that can be examined using qualitative methods (Rossi et al., 2019; Ngai et al., 2022). Accordingly, we performed a mixed-methods evaluation to investigate the effectiveness of the HKBM (Community) Pilot Program, operated as an intervention, on the improvement of NEY’s career-related competencies, as well as to explore the stakeholders’ (e.g., NGO leaders, social workers, parents, employers, and NEY) subjective perceptions and opinions on the implementation of the HKBM (Community).

## 3. Method

### 3.1. Participants and data collection

To evaluate the effectiveness of the HKBM (Community) Pilot Program, we employed a pretest–posttest quasi-experimental design in our study. The pilot group consisted of NEY who participated in the HKBM (Community) Pilot Program implemented by the six local NGOs. Each participating NGO was also asked to invite other NEY who had neither joined the pilot program nor received the CLD interventions to participate in the comparison group. The participants in the pilot group and their counterparts in the comparison group were invited to complete a self-report questionnaire at the pretest and posttest, which were separated by an interval of 4 months. Ultimately, we recruited a youth sample of 449 participants 13–29 years old (*M* = 19.33, *SD* = 3.341) to complete a self-report questionnaire: 289 in the pilot group and 160 in the comparison group. The participants were informed about the purpose and procedures of the study, and parental consent was obtained for all participants less than 18 years old. The method employed in our study was assessed and approved by an ethical review committee before its administration.

Table 1 shows the sociodemographic characteristics of the participants. Regarding participants in the pilot group, more than half were young women (52.6%), and the mean age was 19.08 years (*SD* = 2.948). Most were of Chinese ethnicity (96.9%), whereas 3.1% were ethnic minorities of South Asian or Southeast Asian descent (e.g., Pakistani, Indonesian, and Filipino). A total of 15.2% of the participants were receiving public assistance from the government. By level of education, the majority (64.9%) had received senior secondary or higher education, whereas most of their parents (93.6% fathers and 94.4% mothers) had received no more than secondary education. Meanwhile, of the 160 participants in the comparison group, 73.1% were young men. Most (99.4%) were of Chinese ethnicity, whereas only 0.6% were ethnic minorities of South Asian or Southeast Asian descent (e.g., Pakistani, Indonesian, and Filipino). A total of 3.7% of the participants received public assistance from the government. By level of education, the majority (76.4%) had received senior secondary or higher education, whereas most of their parents (82.3% fathers and 89.5% mothers) had received no more than secondary education.

**Table 1:** Profile of Participants in the Quantitative Study.

Variables	Pilot group (*N* = 289)	Comparison group (*N* = 160)
	Mean/Percentage	Mean/Percentage
Age (years)	19.08 (*SD* = 2.948)	19.77 (*SD* = 3.924)
Gender		
Male	47.4%	73.1%
Female	52.6%	26.9%
Ethnicity		
Chinese	96.9%	99.4%
Non-Chinese	3.1%	0.6%
Father Education		
Primary level or below	19.4%	17.6%
Junior secondary level or equivalent	46.0%	23.5%
Senior secondary level or equivalent	28.1%	41.2%
Associate degree or equivalent	1.4%	11.8%
Bachelor or above	5%	5.9%
Mother Education		
Primary level or below	28.5%	21.9%
Junior secondary level or equivalent	41.7%	28.6%
Senior secondary level or equivalent	24.3%	39.0%
Associate degree or equivalent	1.4%	5.7%
Bachelor or above	4.2%	4.8%
Educational Level		
Primary level	0.4%	1.9%
Junior secondary level	34.7%	21.9%
Senior secondary level	45.8%	8.8%
Diploma or certificate courses	13.7%	64.4%
Higher diploma or associate degree	2.9%	1.3%
Bachelor or above	2.5%	1.9%
Receiving public assistance		
Yes	15.2%	3.7%
No	84.8%	96.3%

Apart from surveys, our study also involved the qualitative method of focus group interviews with NGO leaders, social workers, NEY, parents, and employers to gather their views on and experiences with the HKBM (Community) Pilot Program. The first and second authors organized and conducted five separate focus groups, which, respectively, consisted of six NGO leaders, eight social workers, eight NEY, four parents, and seven employers. Table 2 shows the background information of the participants of the five focus groups.

**Table 2:** Background information of focus group participants.

Code	Stakeholder	Gender	Age	Educational Level
Participant 1	NGO Leader	Male	40–49	Bachelor or above
Participant 2	NGO Leader	Female	50–59	Bachelor or above
Participant 3	NGO Leader	Female	40–49	Bachelor or above
Participant 4	NGO Leader	Male	40–49	Bachelor or above
Participant 5	NGO Leader	Female	40–49	Bachelor or above
Participant 6	NGO Leader	Female	40–49	Bachelor or above
Participant 7	Social Worker	Female	30–39	Bachelor or above
Participant 8	Social Worker	Male	30–39	Bachelor or above
Participant 9	Social Worker	Female	40–49	Bachelor or above
Participant 10	Social Worker	Male	30–39	Bachelor or above
Participant 11	Social Worker	Female	40–49	Bachelor or above
Participant 12	Social Worker	Female	30–39	Bachelor or above
Participant 13	Social Worker	Male	30–39	Bachelor or above
Participant 14	Social Worker	Male	30–39	Bachelor or above
Participant 15	NEY	Male	20–29	Associate degree
Participant 16	NEY	Male	20–29	Senior secondary level
Participant 17	NEY	Female	20–29	Senior secondary level
Participant 18	NEY	Female	10–19	Junior secondary level
Participant 19	NEY	Female	20–29	Senior secondary level
Participant 20	NEY	Male	10–19	Junior secondary level
Participant 21	NEY	Male	20–29	Associate degree
Participant 22	NEY	Male	20–29	Senior secondary level
Participant 23	Parent	Female	40–49	Junior secondary level
Participant 24	Parent	Male	40–49	Primary level
Participant 25	Parent	Female	40–49	Junior secondary level
Participant 26	Parent	Male	40–49	Junior secondary level
Participant 27	Employer	Male	20–29	Associate degree
Participant 28	Employer	Male	30–39	Bachelor or above
Participant 29	Employer	Male	50–59	Bachelor or above
Participant 30	Employer	Male	40–49	Bachelor or above
Participant 31	Employer	Male	30–39	Secondary level
Participant 32	Employer	Female	30–39	Secondary level
Participant 33	Employer	Female	30–39	Secondary level

The focus group interviews were conducted in Cantonese and lasted for approximately an hour. Guiding questions for NGO leaders and social workers included “How do you see the impact of the HKBM (Community) on social workers?” and “How did the HKBM (Community) facilitate social workers’ effectiveness in helping youth?” By contrast, guiding questions for NEY included “What have you learned from the activities that you have attended?” and “Which of those activities impressed you the most? Why?” Meanwhile, guiding questions for parents included “Have you received parental services provided by NGOs?” and “Did the services help you to understand the CLD of your children?” Last, guiding questions for employers included “What type of workplace learning does your company provide for youth referred by NGOs?” and “What drives your company to provide workplace learning to youth?” Ethical approval was obtained from an ethical review committee prior to the commencement of the interviews.

### 3.2. Measures

As mentioned in the Introduction, the effectiveness of the HKBM (Community) Pilot Program was assessed by two career-related measures in our study: YCDC and CLDH.

#### 3.2.1. Youth career development competency

YCDC, encompassing four dimensions—engagement, self-understanding, career and pathway exploration, and planning and career management—was measured using 17 items developed by Ngai et al. (2021). Items included “Continuously participated in my selected activities and new experiences” (i.e., engagement), “Maintained a sense of hope in achieving career and life development aspirations and goals” (i.e., self-understanding), “Compared different career and life development pathways according to personal and environmental factors” (i.e., career and pathway exploration), and “Coped with future career and life development transitions and changes and the stress involved” (i.e., planning and career management). NEY participants were asked to rate their ability to perform a list of activities in the past month, each on a 5-point scale ranging from 1 (*not confident at all*) to 5 (*highly confident*). Ngai et al. (2021) verified the validity of the YCDC Scale by using a sample of 682 individuals aged 15–29 years in Hong Kong. Their exploratory factor analysis revealed four competence factors (i.e., engagement, self-understanding, career and pathway exploration, and planning and career management; total variance explained = 78.95%), while their confirmatory factor analysis revealed a good model fit (CFI = 0.96, TLI = 0.95, RMSEA = 0.06, 90% CI [0.05, 0.07], SRMR = 0.03) and good factor loadings (0.78–0.91). Their study also showed that the YCDC Scale was significantly and positively correlated with other career-related and psychosocial outcomes (e.g., career outcome expectancy, career adaptability, civic engagement, and social contribution), which indicates good concurrent validity. Overall, their results demonstrate that the YCDC Scale is a valid tool for measuring career development competence among youth in the Hong Kong context (Ngai et al., 2021). In this study, YCDC was measured at both baseline and follow-up surveys. The reliability estimates of the scale at baseline and in the follow-up surveys for the pilot group, comparison group, and the full sample were high (see Table 3), with Cronbach’s alphas ranging from 0.845 to 0.970.

**Table 3:** Reliability estimates of career-related measures.

Evaluation scale	Reliability (Cronbach’s Alpha)					
	Pilot group		Comparison group		Overall sample	
	Pre	Post	Pre	Post	Pre	Post
YCDC	0.953	0.953	0.955	0.970	0.953	0.962
Engagement	0.906	0.893	0.885	0.930	0.900	0.914
Self-understanding	0.916	0.900	0.879	0.933	0.905	0.919
Career and pathway exploration	0.915	0.903	0.927	0.958	0.919	0.927
Planning and career management	0.870	0.845	0.927	0.925	0.890	0.881
CLDH	0.939	0.940	0.938	0.941	0.939	0.943
Career and life development agency	0.894	0.865	0.876	0.870	0.889	0.875
Career and life development pathways	0.934	0.936	0.947	0.953	0.938	0.953

#### 3.2.2. Career and life development hope

CLDH, encompassing two dimensions—career and life development pathways and career and life development agency—was measured using 20 items developed by Ngai et al. (2022). Items included “Had encounters with the business sector to understand the industry” and “Took initiatives to set career and life development goals and plans” (career and life development pathways), as well as “Was confident to make career choices that suit me” and “Maintained positive work attitudes and life values” (career and life development agency). NEY participants were asked to appraise their personal ability to perform a list of activities in the past month, each measured on a 5-point scale ranging from 1 (*never*) to 5 (*always*). Ngai et al. (2022) tested the validity of the CLDH Scale by using a sample of 1998 individuals aged 13–29 years in Hong Kong. Their exploratory factor analysis revealed two component factors (i.e., career and life development pathways and career and life development agency; total variance explained = 63.08%), while their confirmatory factor analysis indicated a good model fit (CFI = 0.93, TLI = 0.93, RMSEA = 0.06, 90% CI [0.06, 0.07], SRMR = 0.04) and good factor loadings (0.61–0.82). They also found that the CLDH Scale was significantly and positively correlated with other career-related and psychosocial outcomes (e.g., youth career development competency, career adaptability, civic engagement, and social contribution), which suggests good concurrent validity. Overall, their results demonstrate that the CLDH Scale is a valid tool for measuring career and life development hope among youth in the Hong Kong context (Ngai et al., 2022). In this study, CLDH was measured at both baseline and follow-up surveys. The reliability estimates of the scale at baseline and in the follow-up surveys for the pilot group, comparison group, and the full sample were high (see Table 3), with Cronbach’s alphas ranging from 0.865 to 0.943.

### 3.3. Data analysis

For the quantitative part of our study, we conducted multivariate repeated-measures analyses of covariance (MANCOVA) by including the scores for YCDC and CLDH at baseline and at follow-up as dependent variables to test the effectiveness of the HKBM (Community) Pilot Program according to two hypotheses:

*H1:* The pilot group is more likely to have an increase in YCDC than the comparison group.

*H2:* The pilot group is more likely to have an increase in CLDH than the comparison group.

The factors in the analyses were group (i.e., pilot or comparison) and time (i.e., baseline and follow-up), while potentially confounding social demographic variables (i.e., gender, level of education, ethnicity, and age) were covariates.

For the qualitative part, by contrast, the interview transcripts were first translated into English by research assistants who are native Cantonese speakers. The transcripts were then carefully checked by the first and second authors to ensure the validity of the translation before using it as the basis of data analysis. Thematic analysis was conducted following the steps described by Braun and Clarke (2006). Initially, all patterned meaning units from the transcripts were screened, identified, grouped, and regrouped, after which related patterns were combined to generate initial themes. The emergent themes were next pieced together to form a comprehensive picture of the impact of HKBM. The themes were subsequently adjusted and readjusted to construct a sensible narrative while ensuring minimal overlap and that each theme was carefully defined to fit the coded data and the entire data set. Last, at least one illustrative extract was selected under each theme to facilitate the writing of the research manuscript and better represent the scope and diversity of the theme’s content. The selection process involved including the extracts that were the most identifiable and reflexive of the themes (Braun and Clarke, 2006).Data were coded and mapped by members of the research team and later cross-checked by the first and second authors.

## 4. Results

### 4.1. Quantitative findings

**Figure 1:**
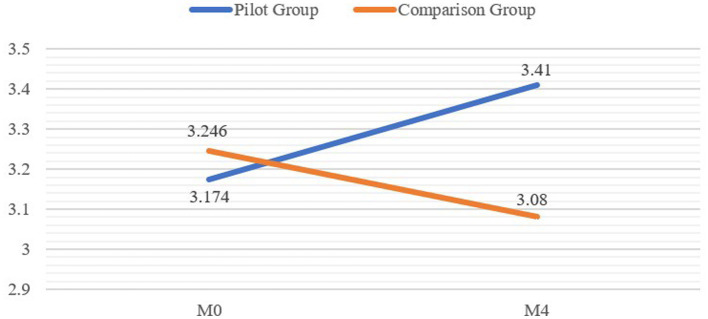
Group comparison of youth career development competency from M0–M4.

The repeated-measures MANCOVA involving baseline and follow-up scores was an omnibus test of the two indicators of career-related outcomes: YCDC and CLDH. Table 4 presented the means and standard deviations of the tested variables at pre- and post-pilot. As shown in Table 5, the analysis revealed small to medium effects (Richardson, 2011). More importantly, we found a significant interaction between time and group for YCDC (*F* = 33.220, *p* < 0.001, partial η^2^ = 0.069; see Table 5), one showing that the increase in YCDC was significantly higher in the pilot group than in the comparison group (see Figure 1). Specifically, YCDC increased from 3.174 to 3.410 in the pilot group but dropped from 3.246 to 3.080 in the comparison group (see Table 4). We found that the interaction between time and group made a significant difference in engagement (*F* = 27.829, *p* < 0.001, partial η^2^ = 0.059), self-understanding (*F* = 18.105, *p* < 0.001, partial η^2^ = 0.039), career and pathway exploration (*F* = 16.011, *p* < 0.001, partial η^2^ = 0.035), and planning and career management (*F* = 17.224, *p* < 0.001, partial η^2^ = 0.037). Taken together, the analysis showed that the increases in the four dimensions of YCDC were significantly higher in the pilot group than in the comparison group, supporting Hypothesis 1.

**Table 4:** Means and standard deviations of the main variables pre- and post-pilot.

Evaluation Variable	Pilot group				Comparison group			
	Pre		Post		Pre		Post	
	Mean	*SD*	Mean	*SD*	Mean	*SD*	Mean	*SD*
YCDC	3.174	0.697	3.410	0.605	3.246	0.639	3.080	0.692
Engagement	3.213	0.833	3.427	0.714	3.280	0.694	3.017	0.770
Self-understanding	3.324	0.815	3.497	0.709	3.303	0.712	3.116	0.801
Career and pathway exploration	3.057	0.775	3.319	0.674	3.174	0.747	3.089	0.768
Planning and career management	3.130	0.806	3.356	0.668	3.230	0.793	3.100	0.754
CLDH	2.911	0.726	3.270	0.634	2.899	0.647	2.928	0.671
Career and life development agency	2.460	0.964	2.956	0.801	2.302	0.829	2.500	0.828
Career and life development pathways	3.154	0.717	3.439	0.654	3.214	0.685	3.154	0.737

**Table 5:** Results of the repeated-measures MANCOVA.

Evaluation variable	Repeated measure MANCOVA								
	Effects of time			Effects of group			Effects of time × group		
	*F*	*p*	*η_p_^2^*	*F*	*p*	*η_p_^2^*	*F*	*p*	*η_p_^2^*
YCDC	1.000	>0.05	0.002	5.635	<0.05	0.012	33.220	<0.001	0.069
Engagement	0.153	>0.05	0.000	8.910	<0.01	0.019	22.009	<0.001	0.067
Self-understanding	0.010	>0.05	0.000	10.192	<0.01	0.022	20.677	<0.001	0.044
Career and pathway exploration	5.171	<0.05	0.011	1.322	>0.05	0.003	20.440	<0.001	0.044
Planning and career management	1.451	>0.05	0.003	1.963	>0.05	0.004	19.962	<0.001	0.043
CLDH	30.238	<0.001	0.063	9.972	<0.01	0.022	21.794	<0.001	0.046
Career and life development agency	50.066	<0.001	0.100	18.818	<0.001	0.040	9.188	<0.01	0.020
Career and life development pathways	9.783	<0.01	0.021	3.563	>0.05	0.008	23.568	<0.001	0.050

**Figure 2:**
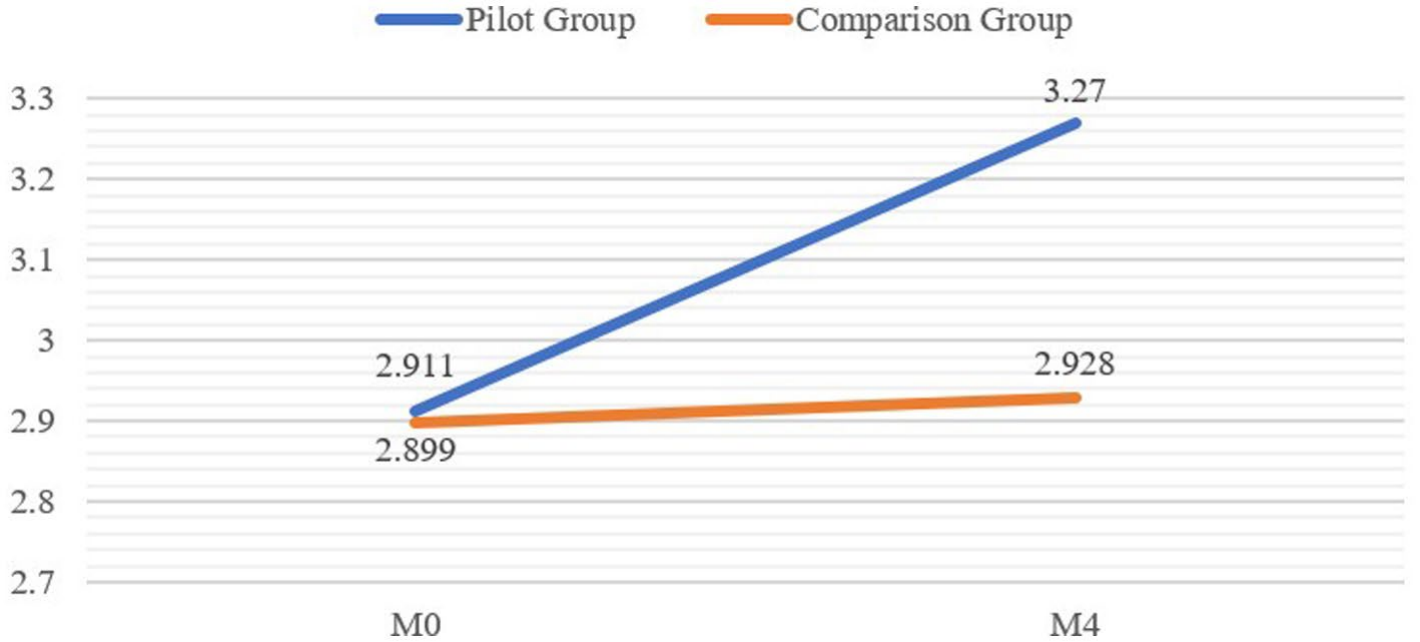
Group comparison of career and life development hope from M0–M4.

Likewise, we found a significant interaction of time and group for CLDH (*F* = 21.794, *p* < 0.001, partial η^2^ = 0.046, see Table 5), one indicating that the increase in CLDH was significantly higher in the pilot group than in the comparison group (see Figure 2). Specifically, CLDH rose from 2.911 to 3.270 in the pilot group but rose only slightly from 2.899 to 2.928 in the comparison group (see Table 4). Furthermore, the interaction between time and group made a significant difference in career and life development agency (*F* = 9.091, *p* < 0.01, partial η^2^ = 0.020) and career and life development pathways (*F* = 23.134, *p* < 0.001, partial η^2^ = 0.040). Taken together, the analysis showed that the increases in the two dimensions of CLDH were significantly higher in the pilot group than in the comparison group, supporting Hypothesis 2.

### 4.2. Qualitative findings

The thematic analysis of qualitative interviews yielded three key themes regarding the stakeholders’ perceived benefits from and barriers to implementing the HKBM (Community). Those themes are summarized and described below.

#### 4.2.1. Providing global standards and guiding principles

The qualitative findings first highlighted that the HKBM (Community) provided global standards and guiding principles for NGO leaders and social workers to review and improve existing services with broadened visions.

##### 4.2.1.1. Providing standards to evaluate existing services

Because each benchmark comprised several measurable criteria, NGO leaders and social workers could easily assess the performance of their existing services based on each criterion and set specific goals for future actions accordingly. Participant 1, an agency leader, stated the following:

It reminds us of which benchmarks we want to achieve when reviewing and planning existing services. (Participant 1).

For that reason, the HKBM (Community) was also recognized by participants as a guiding framework. As Participant 5, who was an NGO leader, pointed out, a “benchmark language” had appeared in the workplace. Her colleagues, whether in her service unit or across service units, could talk about commonly recognized standards, which facilitated the collaboration of the entire NGO.

##### 4.2.1.2. Adopting a comprehensive approach

NGO leaders and frontline social workers embraced the HKBM (Community) as a set of guiding principles also because they benefited from its comprehensive approach to CLD services. In particular, they were inspired to shift the focus of youth services from employment to multiple pathways, thereby crossing boundaries of thinking about the school-to-work transition and revealing new possibilities for practice. Participant 2 and Participant 6, both NGO leaders, stated the following:

We used to be employment-focused, but we overlooked that they [NEY] might want to go back to school or try different pathways. (Participant 2).

My colleagues all felt that the HKBM (Community) inspired us to envision multiple pathways to employment.... It has expanded our horizons and guided us to try out more possibilities. (Participant 6).

#### 4.2.2. Underscoring a youth-focused perspective

The qualitative findings also revealed a youth-focused perspective in the HKBM (Community) Pilot Program. In other words, NEY were generally seen as active agents who were able to develop themselves instead of passive recipients of youth services provided by NGOs. Beyond that, NEY enjoyed a more egalitarian relationship with social workers and parents.

##### 4.2.2.1. Enhancing self-knowledge of youth and affording them meaningful encounters with the workplace

The NEY participants generally praised the pilot program for enabling them to explore their interests, develop better self-understanding, and enhance their career competency. For example, Participant 21, an NEY who participated in various activities conducted by an NGO, remarked that his social worker has helped him explore his interests and get an internship:

After learning about my interests, my social worker told me that a company would offer me an internship for a few months. (Participant 21).

Often, youth encounter difficulties with identifying their strengths or articulating their values and skills. The pilot program facilitated youth in identifying and using their strengths while simultaneously motivating them to develop the skills that they need to succeed. Participant 16, an NEY participant in the pilot program, stated the following:

I’ve accumulated work experiences here. I’ve also gotten subsidies. I’ve become more proactive in my career and life development and have learned different skills. (Participant 16).

CV360 and the career fair have helped me understand my values and demonstrate my strengths. (Participant 16).

The pilot program also allowed youth to interact with business stakeholders such as employers and employees, understand the various work environments and workplace standards, and pursue their CLD interests through experiential learning. With improved self-knowledge, NEY were more likely to stick with a clear career direction and obtain more stable employment experience. The following quotation from Participant 27, who provided NEY with encounters at a boxing gym, is a typical example:

We started by providing them [NEY] with encounters at our gym. Afterward, they took internships, transitioned into part-time coaching, and eventually became full-time licensed coaches. We all felt that it was a meaningful journey. Their loyalty to our gym was greater than that of openly hired coaches. (Participant 27).

Added to that, the participants appreciated the CLD tools provided to them, which could be used to scaffold opportunities for NEY to reflect positively on their workplace encounters and better understand themselves. For instance, NEY participants were guided to complete an online form called CV360® to generate their all-round profile (CLAP@JC, 2022c). CV360® differs from a traditional resume in that it presents not only one’s academic qualifications, awards, and relevant work experiences but also their broader career-related competencies by indicating the values, attitudes, skills, and knowledge articulated from their career and life experience. Participant 16, who benefited from using CV360®, reflected on his experience and stated:

I have accumulated wonderful work experience.... CV360® has helped me to understand my values and myself and to demonstrate my strengths. (Participant 16).

##### 4.2.2.2. Stressing a more egalitarian relationship

Nearly all participants agreed that the HKBM (Community) Pilot Program has made the relationship between NEY with social workers and parents far more equal than it used to be. A typical example is that NEY participants were treated as co-creators of activities in the process of participation. Instead of receiving the services passively, NEY were given a chance to design and implement CLD activities together with social workers. Both parties were pleased to see a more egalitarian relationship develop between them. Participant 8, a social worker who co-created activities with NEY, recalled:

We asked about the young people’s career-related interests or their interests in different types of activities in advance. Then we organized the activities based on their interests. It wasn’t only about providing services. It was also about the relationship between social workers and service users, which has become more egalitarian. (Participant 8).

In addition, Participant 17, an NEY participant who designed and implemented an activity titled “CeleBaby” for young mothers, shared how she has enjoyed an egalitarian relationship with social workers while co-creating the event:

CeleBaby is a co-creation with social workers. I’m involved in the whole process, from initial planning to designing and organizing activities and preparing supplies. (Participant 17).

Beyond that, parent–child relationships also improved. Parents reported that they learned how to understand their children’s needs from their perspectives and without imposing their expectations on their children. As a result, they were more willing to listen to NEY and provide more care and support according to their needs. As Participant 23, who supported her daughter through her school-to-work transition, indicated:

[From the pilot program] I learned that when my daughter encounters difficulties, I should talk to her with an open mind. Parents need to listen to them [NEY] patiently and give advice as friends.... As a mother, all I can do is support and care for my daughter from her point of view. (Participant 23).

It is worth noting that parents in Hong Kong envision that their children will be admitted to a university or further their education after finishing high school and believe that doing so is the only path that their children should pursue. For that reason, most youth in Hong Kong, in order to please their parents, view success as getting into higher education only. Youth unable to pursue higher education thus see themselves as failures and have no sense of direction for their careers. At the same time, their parents become disheartened, which causes the parent–child relationships to deteriorate. Under the influence of this unique sociocultural background, most young people in Hong Kong tend to conceive their career prospects too narrowly (Ngai et al., 2022). Against that backdrop, parents have an important role to play in supporting their children’s CLD if they can provide space and opportunities for their children to identify their interests and strengths, explore multiple career pathways, and develop a greater capacity to aspire (Ngai et al., 2021).

#### 4.2.3. Current barriers and challenges

As a pilot program, the HKBM (Community) Pilot Program presents stakeholders with challenges and barriers in its full application. Qualitative findings revealed such challenges and barriers faced by NEY, NGO leaders, social workers, and parents as they participated in the program.

##### 4.2.3.1. Initially not grasping the entire HKBM (Community) framework

NGO leaders and social workers mentioned struggling to grasp the entire HKBM (Community) framework at an early stage, which hindered their implementation of the approach. For example, they were confused about whether they should achieve the 10 benchmarks simultaneously or in stepwise fashion. Fortunately, after the BM Facilitators clarified the rationale of the HKBM (Community), NGO leaders and social workers understood that they could formulate and implement actions to achieve benchmarks based on their organizational needs and pace. As one NGO leader recalled:

Initially, we did not have a clear idea of the HKBM (Community).... We were confused about whether we had to achieve so many benchmarks simultaneously. (Participant 2).

##### 4.2.3.2. Limiting workplace exposure to some industries

Although NEY participants appreciated the provision of encounters with the workplace, they found that the available industries were limited in type, which hindered them from exploring other career pathways. Therefore, the participating NEY hoped that the NGOs would cover more industries in the future, even marginalized and/or unpopular ones. Participant 16, for example, was impressed by a career-related workshop in which the organizer invited people from both the beauty and the funeral industries to share their work experiences. To him, creativity in workplace exposure mattered. He went on to say the following:

There have been very different industries in the Hong Kong market. Maybe they [organizers] could invite professionals from different industries to talk about their career experiences in order to enrich or refresh our understanding of existing careers. (Participant 16).

##### 4.2.3.3. Having inaccessible CLD-related information

Parents expressed concerns with their ability to access CLD-related information. According to the HKBM (Community), parental support was a key component of making quality CLD services for NEY. However, the participants said that most parents could only receive CLD-related information *via* their children, primarily because the information sharing between organizers and parents has not been well established. In response, a parent in the focus group stated:

I wonder whether CLD-related information can be spread to different groups through instant messaging applications. I think that would be more effective than the traditional promotion because we parents use mobile phones, too. (Participant 25).

## 5. Discussion

The HKBM (Community) Pilot Program was implemented as the world’s first CLD intervention program promoting a benchmark approach in communities. As a leading pilot program focusing on NEY’s problems in Hong Kong, where the unemployment rate among youth far exceeds the rate in the general population (Ngai et al., 2022), the program’s significance was found to lie in its aims for helping NEY become active navigators and agents of their pathways to desired life and career goals as well as empowering the stakeholders involved (e.g., social workers, employers, and parents) to adopt the benchmark approach to enhance the quality of their existing CLD services for NEY. The primary purpose of our study was to examine the effectiveness of the HKBM (Community) Pilot Program. We used a pretest–posttest quasi-experimental design to evaluate the effects of the program in enhancing NEY’s career-related competencies—namely, YCDC and CLDH. We also adopted the qualitative method of focus group interviews to explore how the pilot program had influenced key stakeholders in the ecosystem of CLD service provisions. In other words, the quantitative component of our research was designed to identify how the pilot program has impacted young people’s CLD, whereas the qualitative component of our research was designed to show how the HKBM (Community) has provided guiding principles for rendering support and service interventions to youth, parents, and service providers. In general, the findings from our quantitative and qualitative research suggest the program’s positive effects on the implementation of the HKBM (Community) and that the service providers can review and enhance their existing CLD services through the HKBM (Community) framework.

The analysis revealed that the development of YCDC differed significantly for the pilot group compared with the comparison group, thereby indicating that the HKBM (Community) Pilot Program had a positive effect on the improvement of NEY’s career development competency. In particular, the results show that increases in engagement, self-understanding, career and pathway exploration, and planning and career management were significantly different between the pilot and comparison groups over time. Those findings further demonstrate the positive effects of the HKBM (Community) Pilot Program on NEY and are consistent with past results showing the effectiveness of youth career intervention programs (e.g., Dixon and Crichton, 2016; Eichhorst and Rinne, 2017; Kluve et al., 2019; Park et al., 2020). However, unlike previous research that focused evaluation on NEY’s employment status, welfare recipiency, or acquisition of career skills (e.g., Alzúa et al., 2013; Choi et al., 2015; Eichhorst and Rinne, 2017), our findings stress the development of different career-related competencies among NEY. Such stress is crucial because career-related competencies are essential to understanding and attaining career development and employability in a rapidly changing, unpredictable labor market (Akkermans et al., 2013). To be specific, our study revealed that NEY who received the intervention activities not only improved their capacity to comprehend career aspirations, engage in self-reflection, and connect self-knowledge with alternative pathways but also enhanced their action-oriented abilities such as engaging in new career-related experiences, seeking opportunities, exploring multiple CLD pathways, setting career and life goals, and managing career transitions. It also revealed that the adoption and implementation of a systematic framework involving the core (i.e., NGO policy), youth-focused, and enabling environment parts of the HKBM (Community) can promote NEY’s career-related competencies in different areas and, in turn, prepare them for a school-to-work transition.

The success of the HKBM (Community) Pilot Program in cultivating NEY with a wide range of career-related competencies might be due to its emphasis on active participation, providing exposure to the world of work, and helping NEY to connect with supportive others (e.g., mentors and community partners) in the community. According to the eighth benchmark, “Meaningful encounters with the workplace” (see Appendix), for example, the six participating NGOs offered NEY opportunities for workplace visits and internship activities through which NEY were encouraged to interact with business stakeholders (i.e., employers and employees), experience real-world work environments, and learn workplace principles by active participation. Such meaningful encounters can provide important enabling environments for NEY to change their state of inactivity and lack of ambition. Moreover, because the HKBM (Community) Pilot Program managed to foster egalitarian youth–adult relationships, NEY may sense that are viewed as active social actors with capacities to handle various challenges in life. As a result, they might be more willing to take the initiative to engage in CLD-related activities and consequently develop different competencies needed for a smooth school-to-work transition. Although our evaluation did not address whether NEY ended up in meaningful education, training, or employment, the preliminary outcomes of the pilot program could be taken as promising signs that NEY were approaching productive pathways toward their desired career and life goals.

The analysis also revealed that the development of CLDH differed significantly for the pilot group compared with the comparison group, thereby indicating that the HKBM (Community) Pilot Program had a positive effect on the enhancement of NEY’s sense of hope for their career and life journeys. To be specific, our results show that the development of the two dimensions of CLDH (i.e., CLD agency and CLD pathways) was significantly different for the pilot group compared with the comparison group over time. That finding suggests that NEY who received CLD services in the HKBM (Community) Pilot Program may achieve a higher level of agency to initiate and sustain the motivation to pursue their desired goals and develop higher-level cognitive and behavioral abilities to generate multiple pathways to achieve goals, which together contributed to a more generic, positive expectation of goal attainment, namely CLDH. Previous studies evaluating youth career intervention programs have often shown young people’s improvement in emotional and psychological aspects (e.g., Sheehy et al., 2011; Beck, 2015; Noh et al., 2018) but left the question open as to whether the psychological and emotional improvement of NEY can be translated into a sense of CLD hope, and subsequently put into practice. In that regard, our results offer evidence of the positive effect of the HKBM (Community) Pilot Program on improving NEY’s sense of hope.

A possible reason for that effect was that the HKBM (Community) Pilot Program broadened the scope of CLD services from an employment-focused orientation to a multiple-pathway orientation, which enabled NEY to break the traditional framework of thinking about the school-to-work transition and thus rekindled their sense of hope about future career and life journeys. Today, career intervention theorists tend to consider the school-to-work transition to be a lifelong process within which multiple forms of transitions can occur (e.g., Nilsson, 2019), and that idea was fully considered in the creation and implementation of the HKBM (Community). The ninth benchmark, “Meaningful encounters with further education opportunities,” for example, stresses that youth should have access to a full range of opportunities for progress, including local and overseas academic and vocational pathways, including higher education, vocational and professional education and training, working holidays, and further education. That benchmark implies that the HKBM (Community) is no longer aimed at NEY’s ultimate employment but at empowering young people to become the navigators of multiple pathways to address difficulties related to their CLD in the future. As a result, the previous hopelessness brought about by the narrowly defined route from education to employment may diminish and, in its stead, be a sense of hope that NEY could gain from the multiple pathways to approaching productive school-to-work transitions.

In general, the qualitative findings show the positive impacts of the HKBM (Community) on the ecosystem of CLD service provision. According to social workers and NGO leaders, having the HKBM (Community) helped them to review and enhance the quality of CLD services with a deeper understanding of what good CLD services look like. According to the social workers and NGO leaders, having the HKBM (Community) helped them to review and enhance the quality of CLD services with a deeper understanding of what good CLD services look like. In the pilot program, social workers do not work alone. On the contrary, they collaborate closely with other stakeholders, including parents and employers, to assist NEY in making informed career choices and accompany them in school-to-work transitions. At the same time, parents and employers receive support from social workers in the form of information about helping NEY to explore their multiple career pathways, unlock their full potential, and assist them in developing positive attitudes and values. In other words, the pilot program fosters cross-sectoral collaboration between social workers, parents, and business partners, which is supported by well-informed supportive measures and tools (e.g., CV360®) to deliver CLD interventions.

However, the qualitative findings also showcase several areas for improvement. First, social workers and NGO leaders initially reported difficulties in comprehending the HKBM (Community). Although the BM Facilitators responded quickly to clarify their confusion, the operational mechanism could be improved to support workers in using the HKBM (Community) more efficiently. One possible means to that end is to integrate the insights and experiences from the pilot program into different supportive measures. For example, participating NGOs in our pilot program have consolidated their good CLD practices into a tool kit with practical guidelines to facilitate the effective implementation of the HKBM (Community) among social workers (CLAP@JC, 2022d). In addition, they have compiled impactful stories on relevant benchmarks of the HKBM (Community) into a book of case studies to promote mutual learning among NGOs and have a greater impact in the community (CLAP@JC, 2022e).

Second, NEY emphasized that encounters with the workplace provided by participating NGOs were limited in type and prevented them from fully exploring their career interests and finding their fit with specific jobs. Their emphasis on career interests and an in-depth understanding of the business world was consistent with what Zhu et al. (2022) also found. For that reason, future implementations of the HKBM (Community) should consider expanding the existing types of business partners involved. For instance, participating NGOs may afford access to enterprises of different sizes, particularly small and medium-sized enterprises, with relatively flexible work environments in which NEY can adapt gradually. Also, the involvement of enterprises could extend from local companies to nonlocal ones, particularly those located in mainland China, to optimize the growing labor market opportunities available there for NEY in Hong Kong. Last, parents underscored the lack of communication channels between them and NGOs, which prevented them from receiving up-to-date information about CLD. A rich body of literature has documented the importance of parents’ influence on young people’s CLD (Alfieri et al., 2015; Xing and Rojewski, 2018; Tsui et al., 2019). Thus, future action plans for promoting the HKBM (Community) need new measures to enhance parents’ engagement. For instance, social workers are advised to devote more effort to approaching NEY’s parents through multiple channels (e.g., conventional media, social media, and NEY themselves) to disseminate and share information and knowledge about CLD. In addition, holding sharing sessions about NEY’s success stories can be another way to advocate for parental support in NEY’s school-to-work transitions. Despite those areas that need strengthening, findings from both our qualitative and quantitative research provide preliminary evidence of the positive impacts of the HKBM (Community) on improving young people’s career-related competencies and promoting the entire ecosystem of CLD service provisions.

## 6. Limitations and implications

Several limitations of our findings should be noted. First, although all of the interaction effects of time and group were significant, the effect sizes only reached small to medium levels (Richardson, 2011). The small to medium effect sizes might have arisen because the interval between the pretest and posttest was only 4 months, which may not have been enough for the NEY to make any substantial improvement in their career-related competencies. Future research could involve more follow-up tests over a longer period, including 1 and/or 5 years later, to test the accumulated impacts and outcomes of the HKBM (Community) Pilot Program on transitions. Second, both the uneven sample size between the pilot group and comparison group and the disproportionate gender balance in the latter reduce the statistical power of our findings. In addition, we could not identify the exact age of the focus group participants because their information was collected by the social workers who helped to recruit the focus group participants. With the promotion of the HKBM (Community) and more NEY participating in the program, future research should consider a randomized trial design to generate more rigorous intervention effects. On top of that, because we tested only NEY’s YCDC and CLDH as two indicators of the outcomes of the pilot program, future evaluation studies should include more transitional outcomes of NEY, including successful transitions to education, training, or employment, as variables to test whether the HKBM (Community) can help NEY to make positive school-to-work transitions, as it claims.

Despite those limitations, our findings contribute to the practice and the theorization of the community-based benchmark approach. The HKBM (Community) Pilot Program is pioneering in implementing the benchmark approach through community-based NGOs to deal with NEY-related issues in Hong Kong. By exhibiting the positive impacts of the HKBM (Community) Pilot Program on NEY using rigorous research methods, our study can be used as solid evidence for advocating the expansion of the existing program in order to engage more NEY from diverse backgrounds. In addition, by investigating pretest and posttest outcomes and introducing a comparison group, our work may encourage evidence-based practice among social workers and local NGO practitioners by applying the HKBM (Community), which has been empirically piloted and evaluated in our study, to provide good CLD interventions and continuously improve the quality of those interventions against global standards (Holman, 2014). Furthermore, our findings may also contribute to theorizing the community-based benchmark approach. Although the creation and implementation of the HKBM (Community) took place in Hong Kong’s unique context, the HKBM (Community)‘s core spirit and fundamental directions, founded on a systematic framework involving the core (i.e., NGO policy), youth-focused, and enabling environment parts, can generate valuable implications to theorize the community-based benchmark approach worldwide. In sum, the preliminary evidence, practical experiences, and potential theoretical contributions generated by our research provide guidance for other countries and regions interested in adopting a community-based benchmark approach to tackle NEY-related issues.

## Data availability statement

The raw data supporting the conclusions of this article will be made available by the authors, without undue reservation.

## Ethics statement

The studies involving human participants were reviewed and approved by Survey and Behavioral Research Ethics Committee of the Chinese University of Hong Kong. Written informed consent to participate in this study was provided by the participants’ legal guardian/next of kin.

## Author contributions

SN, C-kC, and Y-hN: conceptualization. SN, C-kC, QZ, L-mW, and Y-hN: methodology. SN, C-kC, QZ, LW, WL, and EY: formal analysis. SN, C-kC, QZ, and HL: investigation. SN: resources, supervision, and funding acquisition. SN, QZ, and WL: data curation. SN, C-kC, QZ, WL, and L-mW: writing—original draft preparation. SN, C-kC, QZ, and EY: writing—review and editing. SN, C-kC, LW, and EY: visualization. SN and H-yT: project administration. All authors have read and agreed to the published version of the manuscript. All authors contributed to the article and approved the submitted version.

## Funding

This project was supported by a grant from the Hong Kong Jockey Club Charities Trust (Grant number: 2020–0111-002).

## Conflict of interest

The authors declare that the research was conducted in the absence of any commercial or financial relationships that could be construed as a potential conflict of interest.

## Publisher’s note

All claims expressed in this article are solely those of the authors and do not necessarily represent those of their affiliated organizations, or those of the publisher, the editors and the reviewers. Any product that may be evaluated in this article, or claim that may be made by its manufacturer, is not guaranteed or endorsed by the publisher.
