# Supplementary material for: Career and life development intervention for non-engaged youth: Evaluating the Hong Kong Benchmarks (Community) Pilot Program
Source: Front Psychol. 2023 Apr 6;14:1117251. doi: 10.3389/fpsyg.2023.1117251 (PMC10115995; doi:10.3389/fpsyg.2023.1117251)
Supplement: Supplementary file 1 [file Data_Sheet_1.pdf]

**Appendix: Hong Kong Benchmarks (Community).**

**Core**

**BM1: A Stable and Visible Career and Life Development Policy**

1.1 Stable CLD policy: Strategy
1.2 Senior management support
1.3 Human resources, budget and infrastructure
1.4 Using stakeholders as resources
1.5 Designated infrastructure
1.6 Regular policy review
1.7 Annual update of action plan
1.8 Visible CLD policy for stakeholders
1.9 Accessible communication channels

**BM2: Professional Competencies and Leadership**

2.1a Leadership: Preparing, implementing and evaluating the CLD annual plan
2.1b Leadership: Leading stakeholders
2.1c Leadership: Communicating with senior management
2.1d Leadership: Team composition review
2.2a Management: Human resources and budget
2.2b Management: Monitoring and supporting members
2.3a Networking and coordination: Frontline NEY service workers
2.3b Networking and coordination: Stakeholders
2.4 CLD team leader: Receives continuing professional development annually
2.5 CLD team: Receives continuing professional development annually
2.6 Initial CLD training received
2.7 Induction for new members
2.8 Annual internal sharing
2.9 Annual external sharing

**Youth Focused**

**BM3: Learning from Multiple Pathways Information**

3.1 Guidance on searching for and processing information about multiple career pathways
3.2 Training for youth on motivating their participation
3.3 Training for youth on enhancing self-understanding
3.4 Three types of diversified CLD programs co-created by youth
3.5 Confident informed decision-making by youth

**BM4: Address the Needs of Each Individual**

4.1a Programs: Personalized advice and support for special target groups
4.1b Programs: Challenging stereotypical thinking
4.2 CLD-oriented exploration programs and tools to identify needs and interests
4.3 CLD tools for building youth’s profiles

**BM5: Youth Engagement and Co-creation**

5.1 Mechanism and supportive measures to facilitate youth's co-creation
5.2 Youth's co-creation of one CLD program annually
5.3 Extending CLD-related connections with adults and peers
5.4a Documenting youth's ideas and co-creation plans
5.4b Collecting youth's wants and needs in CLD programs
5.4c Responding to youth's wants and needs in CLD programs

**BM6: Career and Life Guidance for Developing Career Roadmaps**

6.1 Mechanism and supportive measures to facilitate youth's co-creation
6.2 Youth's co-creation of one CLD program annually
6.3 Extending CLD-related connections with adults and peers
6.4a Documenting youth's ideas and co-creation plans
6.4b Collecting youth's wants and needs in CLD programs
6.4c Responding to youth's wants and needs in CLD programs

**Enabling Environment**

**BM7: Linking Youth Service Plans to Career and Life Development**

7.1 Integrating CLD-related concepts and practices into regular activities and service plans
7.2 Subject-embedded CLD: Career readiness-related information
7.3 Subject-embedded CLD: Concepts of career roadmaps
7.4 Subject-embedded CLD: Connection with workplace resources

**BM8: Meaningful Encounters with the Workplace**

8.1 Workplace learning activities co-created by youth
8.2 Debriefing or follow-up with youth
8.3 Introducing youth's characteristics and CLD-related needs to employers
8.4 Providing opportunities for youth's interaction with employees or employers
8.5 Tools to debrief youth on their reflections

**BM9: Meaningful Encounters with Further Education Opportunities**

9.1 Programs for continuing and higher education
9.2 Meaningful visits or tours for youth
9.3 Opportunities for youth to interact with CLD practitioners
9.4 Guidance on linking interactions with CLD-related goal setting

**BM10: Parent Engagement and Support**

10.1 Parental engagement coordinated by staff
10.2 Providing services for parents to foster an environment of mutual support
10.3 Providing information about multiple career pathways to parents
